# Metabolic engineering of *Escherichia coli* for poly(3-hydroxybutyrate) production via threonine bypass

**DOI:** 10.1186/s12934-015-0369-3

**Published:** 2015-11-20

**Authors:** Zhenquan Lin, Yan Zhang, Qianqian Yuan, Qiaojie Liu, Yifan Li, Zhiwen Wang, Hongwu Ma, Tao Chen, Xueming Zhao

**Affiliations:** Key Laboratory of Systems Bioengineering (Ministry of Education), Tianjin University, Tianjin, 300072 China; SynBio Research Platform, Collaborative Innovation Center of Chemical Science and Engineering (Tianjin), School of Chemical Engineering and Technology, Tianjin University, Tianjin, 300072 China; Edinburg-Tianjin Joint Research Centre for Systems Biology and Synthetic Biology, Tianjin University, Tianjin, 300072 China; Key Laboratory of Systems Microbial Biotechnology, Tianjin Institute of Industrial Biotechnology, Chinese Academy of Sciences, Tianjin, 300308 China; Department of Biochemical Engineering, School of Chemical Engineering and Technology, Tianjin University, Nankai District, 92 Weijin Road, Tianjin, 300072 China

**Keywords:** Genome-scale metabolic network, Threonine bypass, Strain optimization, Poly(3-hydroxybutyrate)

## Abstract

**Background:**

Poly(3-hydroxybutyrate) (PHB), have been considered to be good candidates for completely biodegradable polymers due to their similar mechanical properties to petroleum-derived polymers and complete biodegradability. *Escherichia coli* has been used to simulate the distribution of metabolic fluxes in recombinant *E. coli* producing poly(3-hydroxybutyrate) (PHB). Genome-scale metabolic network analysis can reveal unexpected metabolic engineering strategies to improve the production of biochemicals and biofuels.

**Results:**

In this study, we reported the discovery of a new pathway called threonine bypass by flux balance analysis of the genome-scale metabolic model of *E. coli*. This pathway, mainly containing the reactions for threonine synthesis and degradation, can potentially increase the yield of PHB and other acetyl-CoA derived products by reutilizing the CO_2_ released at the pyruvate dehydrogenase step. To implement the threonine bypass for PHB production in *E. coli*, we deregulated the threonine and serine degradation pathway and enhanced the threonine synthesis, resulting in 2.23-fold improvement of PHB titer. Then, we overexpressed *glyA* to enhance the conversion of glycine to serine and activated transhydrogenase to generate NADPH required in the threonine bypass.

**Conclusions:**

The result strain TB17 (pBHR68) produced 6.82 g/L PHB with the yield of 0.36 g/g glucose in the shake flask fermentation and 35.92 g/L PHB with the yield of 0.23 g/g glucose in the fed-batch fermentation, which was almost 3.3-fold higher than the parent strain. The work outlined here shows that genome-scale metabolic network analysis can reveal novel metabolic engineering strategies for developing efficient microbial cell factories.

**Electronic supplementary material:**

The online version of this article (doi:10.1186/s12934-015-0369-3) contains supplementary material, which is available to authorized users.

## Background

Due to increased concerns on resource depletion and environmental issues, bioplastics that can be produced from renewable resources have attracted great attention [1, 2]. Polyhydroxyalkanoates (PHAs), which were accumulated as carbon reserve materials in response to the availability of excess carbon source by many microbial species, are promising carbon–neutral biodegradable substitutes for petroleum-derived synthetic polymers [3, 4]. Due to their unique properties such as biodegradability, biocompatibility, water resistance, and oxygen impermeability, PHAs can be used in many fields, such as plastics, medical implants and drug delivery carriers [3]. PHAs can be synthesized and produced by chemical procedures or microbial fermentation. The microbial fermentation for PHA production leads to much higher molecular weights than that of the chemical procedures [5]. Poly(3-hydroxybutyrate) (PHB) is the most widespread and best-characterized member of PHA and is synthesized by various microorganisms in nature [6].

**Fig. 1:**
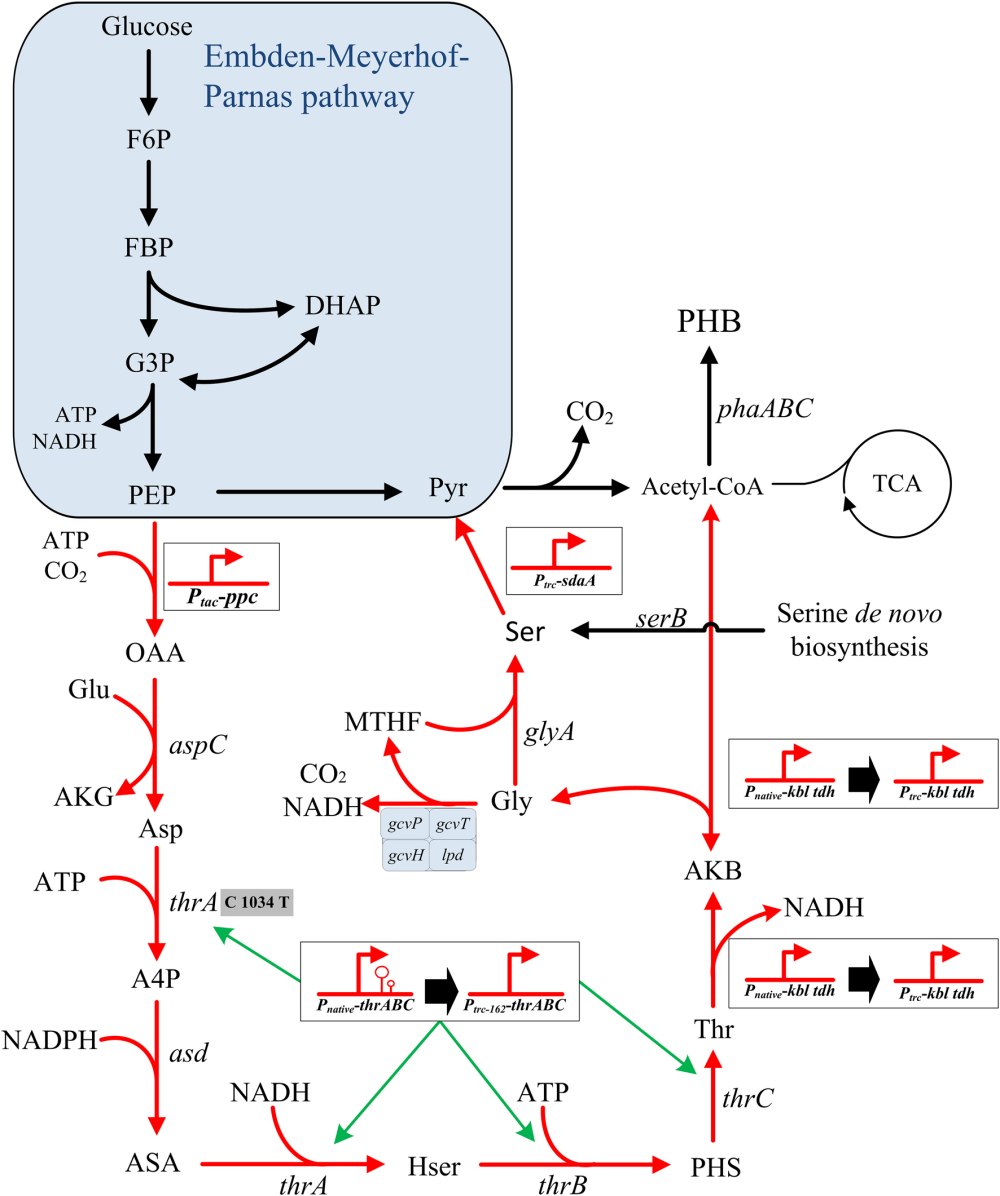
The optimal pathway for threonine production calculated from *iJO1366* model and the related genetic engineering targets used in this study. *Red lines* indicate reactions in the threonine bypass. Through threonine bypass, the theoretical mole yield of PHB was increased from 1 to 1.26. The metabolites are: *F6P* fructose 6-phosphate, *FBP* fructose 1,6-bisphosphate, *GAP* d-Glyceraldehyde 3-phosphate, *DHAP* dihydroxyacetone phosphate, *PEP* phosphoenolpyruvate, *Pyr* pyruvate, *OAA* oxaloacetate, *Asp* aspartate, *A4P* aspartyl-4-phosphate, *ASA* aspartate semialdehyde, *Hser* homoserine, *PHS* homoserine phosphate, *Thr* threonine, *AKB* 2-amino-3-ketobutyrate, *Gly* glycine, *Ser* serine, *MTHF* methylene-tetrahydropteroylpolyglutamates, *Glu* glutamate, *AKG* 2-oxoglutarate

The microbial production of PHB utilizes various species of *Ralstonia eutropha*, *Alcaligenes latus*, *Bacillus* spp., *Azotobacter vinelandii*, and *Pseudomonas* sp. [3]. The underlying biosynthetic route is encoded by the *phbCBA* operon (Fig. 1). PHB is synthesized through the condensation of two acetyl-CoA molecules into acetoacetyl-CoA, its reduction to hydroxybutyryl-CoA, and the polymerization of the latter [6, 7]. Recombinant *E. coli* strains harboring the *R. eutropha* PHA biosynthesis genes have also been used for the production of PHB [8–10].

Economic evaluation of the process for the production of PHB suggested that the major contributor to the overall PHB production cost was carbon substrate cost (up to 50 % of the total cost) [11]. The cost of substrate is the main economical barrier for the application of PHA toward consumer plastics. Therefore, a range of metabolic engineering studies have been carried out with the objective to improve the product yield and produce PHB from cheap carbon source (whey, hemicellulose) by using recombinant *E. coli* [12]. The PHB yield was increased by 12.3 % in the phosphoglucose isomerase (*pgi*) mutation *E. coli* [13]. The strategy of reducing mix acid accumulation was also employed to enhance the PHB production and the yield was increased by 4.3-fold [14]. In recombinant *E. coli*, an *arcA* mutation was used for PHB accumulation under microaerobic conditions using glucose or glycerol as a carbon source [15, 16].

Despite many studies have been carried out to improve the PHB yield, there has been no report of a PHB over-producer that can compete with the theoretical yield in the past years [11]. A possible explanation might be that the local, rather than systems-oriented, strategies used in previous approaches may have limited strain improvement [17]. Computational strain design procedures in the past have predicted interventions away from target pathways that propagate carbon flux through the stoichiometry to further boost yield. Flux balance analysis (FBA) has been used to predict genetic interventions for strain redesign in *E. coli*. In the present study, we presented a novel engineering strategy discovered through pathway analysis of genome scale metabolic network of *E. coli*. A new pathway, termed Threonine Bypass, was found to be able to convert one mole phosphoenolpyruvate (PEP) and one mole CO_2_ to two mole acetyl-CoA theoretically, by utilizing the excess reducing power and ATP generated in PHB production. We engineered this pathway in *E. coli* by activating the Threonine Bypass, and subsequently PHB yield was significantly increased using glucose as carbon source. The final strain TB17 (pBHR68) produced 6.82 g/L PHB with the yield of 0.36 g PHB/g glucose in shake flask fermentation, and 35.92 g/L in fed-batch fermentation. As acetyl-CoA is the precursor for many biological products, the engineering of threonine bypass could be a general strategy for strain optimization to improve product yield.

## Results and discussion

### Discovery of threonine bypass through metabolic network analysis

**Fig. 2:**
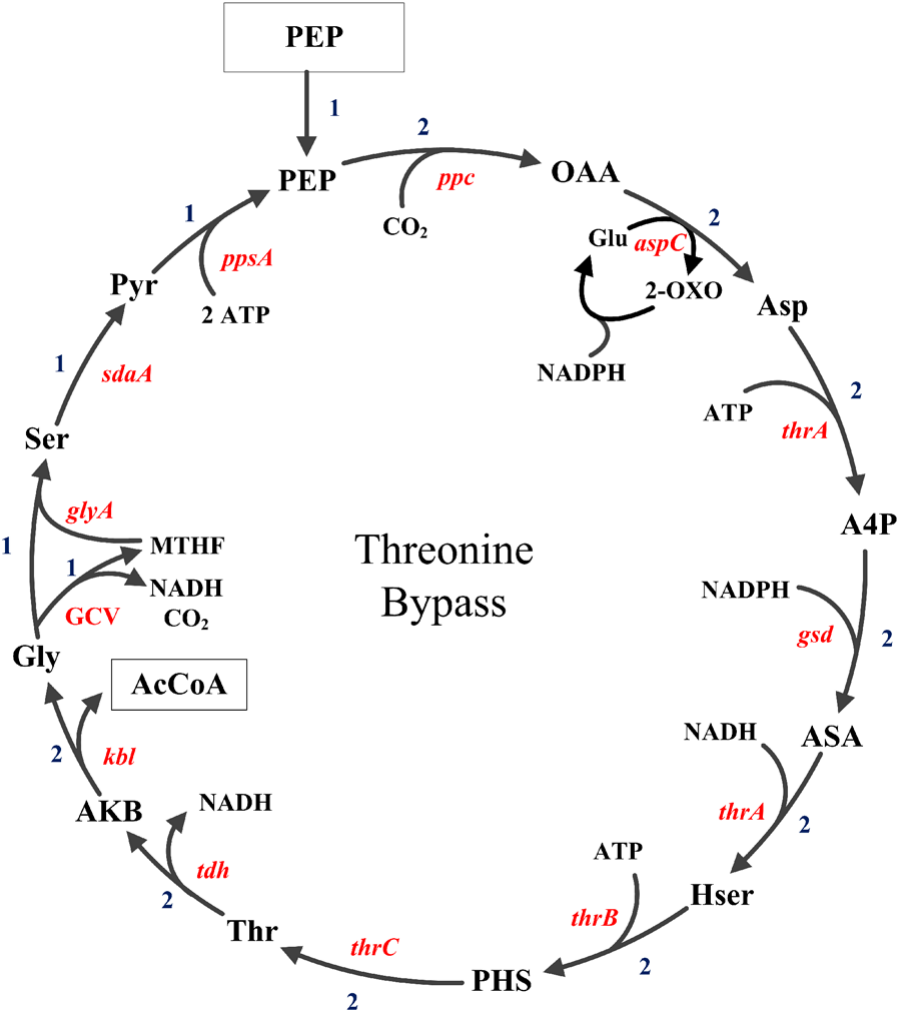
The scheme of the entire threonine bypass from PEP. Two molecules of AcCoA were produced from PEP by CO_2_ fixation at the expense of reducing power and energy. The *numbers* indicate the relative fluxes to PEP input. The metabolites are: *PEP* phosphoenolpyruvate, *Pyr* pyruvate, *OAA* oxaloacetate, *Asp* aspartate, *A4P* aspartyl-4-phosphate, *ASA* aspartate semialdehyde, *Hse* homoserine, *PHS* homoserine phosphate, threonine, *AKB* 2-amino-3-ketobutyrate, *Gly* glycine, *Ser* serine, *Thr* *MTHF* methylene-tetrahydropteroylpolyglutamates, *Glu* glutamate, *2-OXO* 2-oxoglutarate

The calculated optimal pathway for PHB production in the *E. coli iJO1366* model is shown in Fig. 1. In addition to the classical pathway from PEP-pyruvate to the PHB precursor acetyl-CoA through pyruvate dehydrogenase complex, there is also a much longer pathway for acetyl-CoA production including PEP carboxylation, threonine synthesis and degradation, serine formation from glycine and serine deamination. We named this pathway threonine bypass as threonine synthesis and degradation are the main parts of the pathway. With this pathway, the theoretical PHB yield is calculated to be 1.26 mol/mol glucose (0.602 g PHB/g glucose), a 26 % increase comparing with 1.0 mol/mol from the classical pyruvate dehydrogenase pathway. To make it clearer, the threonine bypass was redrawn as a cycle pathway starting from PEP and depicted in Fig. 2. The overall reaction equation of the cycle can be written as:

$$ \begin{aligned} & {\text{PEP}} \,+\, {\text{CO}}_{ 2} \,+\, 2   {\text{CoA}}\, + \,4 {\text{NADPH}} \,+\, {\text{NAD}}^{ + } \,+\, 6   {\text{ATP}} \to \hfill \\ & 2 {\text{Acetyl}}{\,-\,} {\text{CoA}} \,+\, 4   {\text{NADP}}^{ + } \,+ \,{\text{NADH}} \,+ \,{\text{6 ADP}} \end{aligned}$$

As NADPH and NADH are regarded as equal in *iJO1366* model due to the existence of transhydrogenase reaction, this equation can be further simplified as:

$$ \begin{aligned}& {\text{PEP}} + {\text{CO}}_{ 2} + {\text{2 CoA}} + {\text{3 NADPH}} + {\text {6 ATP}}  \to \hfill \\ & 2 {\text{Acetyl}} - {\text{CoA}} + {\text {3 NADP}}^{ + } + {\text{ADP}} \end{aligned}$$

This indicates that through threonine bypass, two acetyl-CoA can be produced from one PEP at the expense of reducing power and energy. This is in contrast to the classical pathway where only one acetyl-CoA is produced from pyruvate due to the carbon loss in the form of CO_2_ generated in the pyruvate dehydrogenase reaction. The overall reaction equation from PEP to acetyl-CoA for the classical pathway is:

$$ {\text{PEP}} + {\text{CoA}} + {\text{NAD}}^{ + } + {\text{ADP }} \to {\text{Acetyl}} - {\text{CoA}} + {\text{NADH}} + {\text{ATP}} + {\text{CO}}_{ 2} $$

Therefore the net produced ATP/NADH from the classical pathway can drive the threonine bypass to produce two acetyl-CoA from one PEP, and thus increase the yield of acetyl-CoA and its derived products like PHB. It should also be noted that the two NADH produced in the EMP pathway (from glucose to PEP) are enough to satisfy the NADH requirement for PHB synthesis from acetyl-CoA. In summary, the threonine bypass can increase the theoretical yield of PHB by making use of the ATP and reducing power generated in the classical PHB production pathway. Based on this analysis results, we decided to construct the threonine bypass pathway in *E. coli* in order to improve PHB yield.

### Degradation of threonine and serine for the PHB production

The successful implementation of the in silico-validated engineering strategy for the PHB production via threonine bypass in *E. coli* requires the modification of several steps, including degradation of threonine and serine, threonine synthesis, conversion of serine from glycine and PEP carboxylation. We first manipulated the degradation pathway of threonine and serine to open up the threonine bypass.

**Fig. 3:**
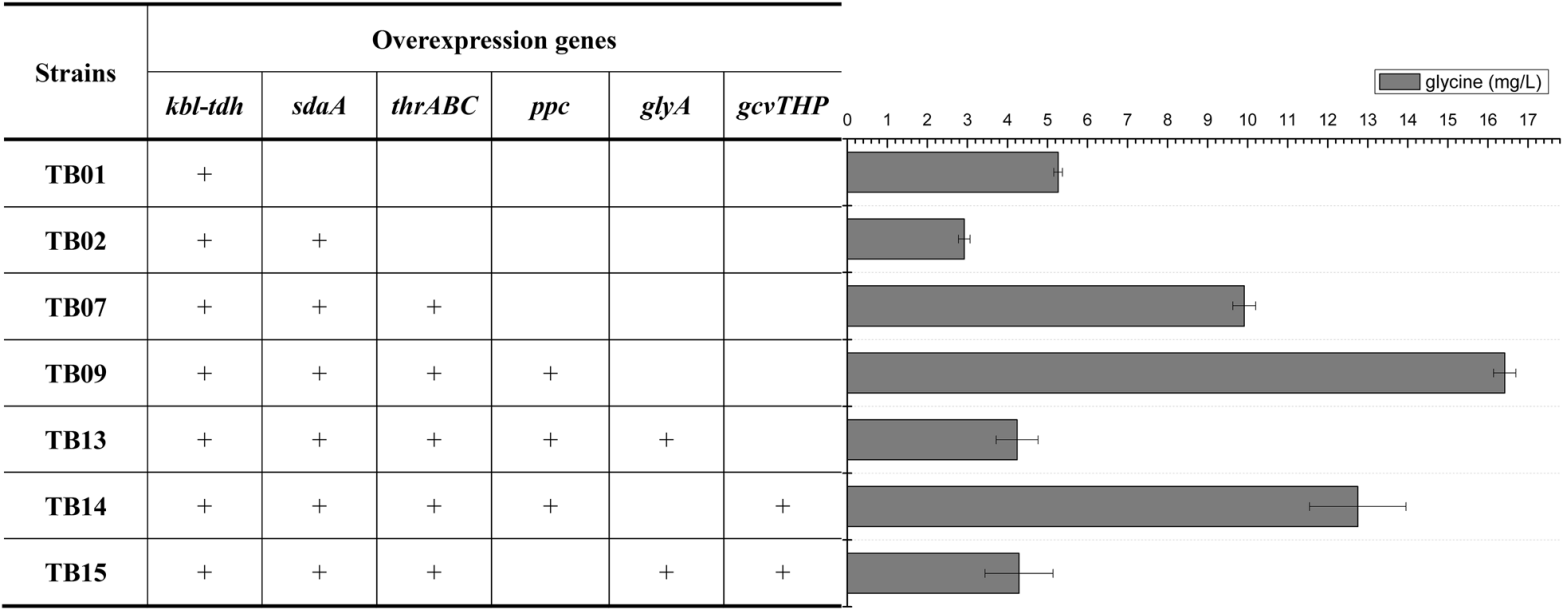
The accumulation of glycine in recombinant *E. coli* strains. Histogram shows the mean of three biological replicates, and *error bars* show standard deviations

As shown in Fig. 1, all enzymes in threonine bypass exist in *E. coli*, but they are often not expressed at the same time due to gene regulation. In culture media lack of threonine, the threonine synthesis pathway is active but its degradation genes are repressed by feedback regulation to avoid energy waste. Therefore, to construct the threonine bypass, we first need to modify the regulation related with the degradation genes. In *E. coli*, there are two pathways for threonine degradation: one uses l-threonine aldolase, the other utilizes threonine dehydrogenase and 2-amino-3-ketobutyrate CoA ligase [18]. Since the threonine aldolase-catalyzed reaction has very low or even undetectable level of activity in a variety of systems, threonine dehydrogenase-catalyzed reaction is recognized as the major route for threonine utilization in *E. coli* [19–21] and it is also the route used in the optimal pathway as shown in Fig. 1. To redirect the carbon flux into the threonine dehydrogenase pathway from threonine, the native promoter of *kbl*-*tdh* operon was replaced with a constitutive promoter *trc* in JM109. The resulting strain TB01 (JM109, P_*trc*_-*kbl*-*tdh*) secreted 5.27 mg/L glycine into the medium and threonine was not detected (Fig. 3), suggesting that threonine was successful redirected into the glycine and acetyl-CoA. Accordingly, the PHB production in strain TB01 (pBHR68) harboring the PHB synthesis genes from *Ralstonia eutropha*, was increased by 2.23-fold with a yield of 0.32 g PHB/g glucose, compared with that in the wild-type strain JM109 (pBHR68) (Table 1).

**Table 1:** PHB accumulation of the recombinant *E. coli* strains

Strains	CDW (g/L)	PHB(g/L)	PHB content (% CDW)	PHB yield (g/g)
JM109 (pBHR68)	3.85 ± 0.085	2.08 ± 0.03	54.0 ± 0.56	0.16 ± 0.01
TB01 (pBHR68)	6.36 ± 0.16	4.64 ± 0.09	73.04 ± 0.48	0.32 ± 0.00
TB02 (pBHR68)	6.52 ± 0.16	5.01 ± 0.17	76.80 ± 0.73	0.34 ± 0.01
TB07 (pBHR68)	7.26 ± 0.05	5.07 ± 0.13	69.84 ± 1.30	0.26 ± 0.00
TB09 (pBHR68)	6.63 ± 0.12	4.63 ± 0.10	69.76 ± 0.88	0.26 ± 0.01
TB13 (pBHR68)	6.95 ± 0.06	5.97 ± 0.14	85.91 ± 1.53	0.30 ± 0.02
TB14 (pBHR68)	5.68 ± 0.28	3.82 ± 0.18	67.24 ± 1.40	0.20 ± 0.01
TB15 (pBHR68)	5.34 ± 0.02	3.72 ± 0.01	69.74 ± 0.51	0.24 ± 0.00
TB17 (pBHR68)	8.64 ± 0.33	6.82 ± 0.33	78.87 ± 0.82	0.36 ± 0.01

The data represent mean values with corresponding deviations from three biological replicates

The last step of threonine bypass is the deamination of serine to pyruvate by serine deaminase, which are encoded by *sdaA*, *sdaB* and *tdcG*, respectively in *E. coli* [22]. Previous studies have indicated that *sdaA*-overexpressing *Corynebacterium glutamicum* could grow in the minimal medium using l-serine as the sole carbon source [23]. Thus, *sdaA* was selected to be overexpressed via swapping the native *sdaA* promoter with a constitutive promoter *trc* in TB01, creating strain TB02. The glycine accumulation by TB02 was 2.92 mg/L, decreased from 5.27 mg/L by TB01 due to the conversion into serine (Fig. 3).

In *E. coli*, there are two pathways for the biosynthesis of serine. The major pathway is the serine de novo synthesis pathway starting from 3-phosphoglyceric acid (PGA) [24]. The second pathway converts threonine to glycine and then serine by assimilation of a C1-unit [25, 26]. In the optimal pathway (Fig. 1), the second pathway is used. Therefore, to divert pathway flux via the optimal pathway, we knocked out the *serB* gene in the serine de novo synthesis pathway to block the serine synthesis from PGA in strains JM109, TB01, and TB02, creating strain TB03, TB04, TB05, respectively. The resulting strains JM109, TB03, TB04 and TB05 were selected in the minimum sodium media plates using glucose as the carbon source with/without serine, glycine and threonine. As shown in Additional file 1: Figure S3a, all the strains, except TB03, could grow in the minimum sodium media without supplement of serine, which indicated that strains TB04 and TB05 could derive serine from glycine for cell growth with the overexpression of *kbl*-*tdh* operon. In *E. coli*, although serine can be synthesized from glycine by glycine hydroxymethyltransferase, glycine converted from threonine was not sufficient for cell growth in strain TB03, which was confirmed by the additional of glycine, serine in the medium (Additional file 1: Figure S3b, c). As shown in Additional file 1: Figure S3d, all the strains, except TB03, could grow well in the medium with threonine, which indicated that threonine could efficient convert into glycine with the overexpression of genes in the threonine degradation pathway.

To evaluate the effect of the overexpression of genes in threonine and serine degradation pathway on PHB production, the PHB biosynthetic pathway was introduced into the engineering strain. As shown in Table 1, the PHB yield of TB02 (pBHR68) (0.34 g PHB/g glucose) was increased by 2.41-fold compared with that of the parent strain. However, compared with TB01 (pHBR68), TB02 (pHBR68) did not significantly improve the PHB content, probably caused by the limitation of conversion of glycine into serine. We also found that the PHB production of the *serB* defective strains was significantly decreased, accompanying little increase of biomass. This phenomenon may be due to the fact that the inactivation of serine de novo synthetic pathway caused serine shortage in the PHB production strain. The improvement of the intercellular glycine pool by deregulation of threonine degradation activated glycine cleavage system for the C1-unit pool. The extracellular amino acids measurement showed that no serine and threonine was accumulated, but small amount of glycine was detected in both TB01 and TB02 (Fig. 3). Therefore, TB02 was chosen as a host for further engineering.

### Combined engineering of threonine synthesis and degradation for the PHB production

It is reasoned that the efficient conversion of l-threonine into acetyl-CoA and glycine requires effective availability of l-threonine. In *E. coli*, threonine is synthesized from l-aspartate via five enzymatic steps. The first step catalyzed by aspartokinase I (encoded by *thrA*) is subject to feedback inhibition by l-threonine [27]. To prevent the feedback inhibition, we mutated base C1034 to T (Ser345Phe) in the *thrA* gene according to the results reported by Lee et al. [28]. Moreover, the repression of the expression of *thrABC* by l-threonine and l-isoleucine is ascribed to the transcriptional attenuation of the operon through *thrL*, which is a leader sequence of *thrABC* operon [29]. To enhance the production of threonine in *E. coli*, transcriptional attenuation was removed by replacing the native promoter of the *thrABC* operon with the 5′-UTR regulatory parts Trc-162 (including a strong constitutive promoter *tac* and a proper RBS), which allows constitutive expression in *E. coli*. The mutated *thrABC* overexpression strains were derived from strain JM109 and TB02, and designated as strains TB06 and TB07, respectively. TB06 accumulated 7.13 mg/L threonine, while glycine was not detected under the same conditions. In contrast, strain TB07 accumulated 9.92 mg/L glycine, a 3.4-fold increase compared with TB02, while threonine was not detected (Fig. 3). These results suggested that threonine could be completely converted to glycine and acetyl-CoA after enhancing the threonine biosynthetic flux and redirecting the carbon flux into glycine via threonine degradation pathway.

As suggested by the in silico design, the threonine bypass can assimilate CO_2_ by the carboxylation reaction of phosphoenolpyruvate to oxaloacetate (OAA) catalyzed by phosphoenolpyruvate carboxylase (PPC, encoded by *ppc* gene). OAA was also an important precursor for threonine and other amino acids in the L-aspartate family. Thus, overexpression of phosphoenolpyruvate carboxylase could enhance the CO_2_ assimilation and benefit oxaloacetate replenishment for threonine production. Moreover, PEP carboxylase has already been targeted to enhance threonine production in *E. coli*, indicating the necessity for adjusting the oxaloacetate pool within the optimal range [30]. To this end, the *ppc* gene was amplified in TB07 by swapping the native promoter with a strong constitutive promoter *tac*, and designated as strain TB09. Strain TB09 accumulated 16.41 mg/L glycine, much higher than TB07 which produced 9.92 mg/L, possibly due to the improvement of threonine synthesis (Fig. 3).

To our surprise, the PHB production of TB07 (pBHR68) was 8 % higher than that of the PPC overexpressing strain TB09 (pBHR68) (Table 1), and the PHB content and yield were almost the same. This phenomenon was probably due to the limitation of the conversion of glycine into serine, which was confirmed by the accumulation of glycine in the medium of TB09 and TB07 (Fig. 3).

### Conversion of glycine into serine for the PHB production

**Fig. 4:**
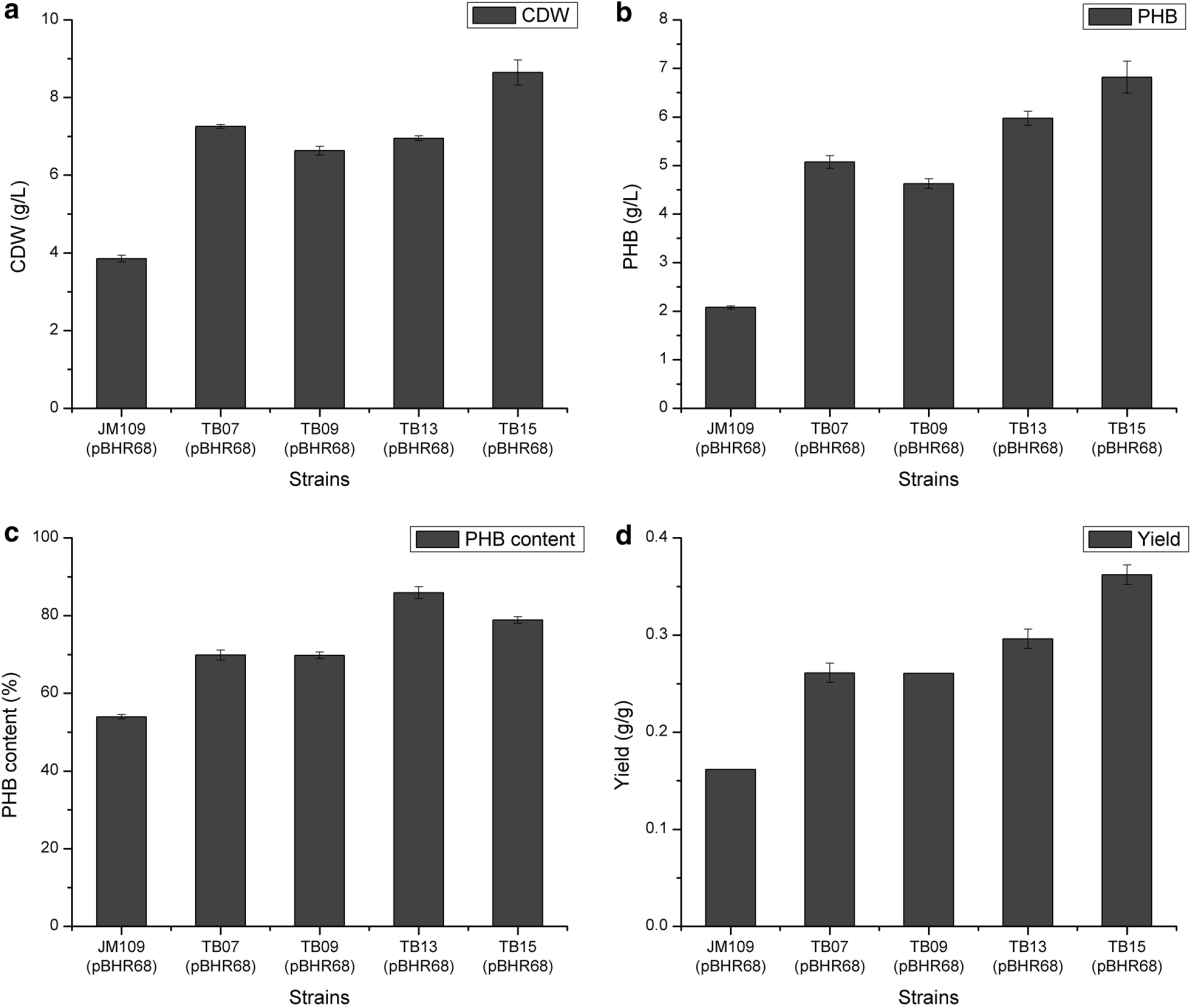
Comparison of CDW (**a**), PHB concentration (**b**), PHB content (**c**), and PHB yield (**d**) in recombinant *E. coli* strains. The data represent mean values with corresponding deviations from three biological replicates

Serine hydroxymethyltransferase (SHMT) catalyzes the reversible interconversion of serine and glycine with tetrahydrofolate serving as the one-carbon carrier [31]. Since glycine accumulation was detected in strains with activated threonine degradation pathway (Fig. 3), we suspected that the conversion of glycine into serine limited the flux of the degradation pathway. Supply of one-carbon unit and SHMT enzyme activity are the major factors affecting the conversion of glycine into serine. In *E. coli*, C1 units are mainly formed from serine cleavage to glycine or from glycine cleavage system (GCV) which produces C1-THF from glycine (Fig. 1). In addition, the conversion of glycine into serine requires the balanced supply of glycine and C1-THF that was also generated from glycine cleavage (Fig. 1). Thus, we inserted the same regulatory part Trc-162 in the upstream of *glyA* gene and *gcv* operon individually or together in TB09, creating strains TB13 (TB09, P_*trc*-*162*_-*glyA*), TB14 (TB09, P_*trc*-*162*_-*gcvTHP*) and TB15 (TB09, P_*trc*-*162*_-*glyA*, P_*trc*-*162*_-*gcvTHP*). As shown in Fig. 3, the accumulation of glycine was decreased after engineering the *glyA* and/or *gcv* operon. The relative gene transcription levels of strain TB13 and TB15 were compared with parent strain JM109 through RT-PCR analysis (Additional file 1: Figure S2), which indicated that all the genes have been successfully overexpressed. The resulting strain TB13 (pBHR68) produced 5.97 g/L PHB with a content of 85.9 % (Fig. 4), and the glycine accumulation of TB13 decreased from 16.42 mg/L (TB09) to 4.24 mg/L (Fig. 3).

However, overexpression of *gcv* operon individually or in combination with *glyA* led to the reduction of PHB production and glycine accumulation in strains TB14 (pBHR68) and TB15 (pBHR68), compared to that of parent strain TB09. This phenomenon may be due to the enhancement of intercellular glycine pool would lead to activate the GCV system and the C1-THF generated from GCV was enough for the conversion of glycine into serine. Previous report indicated that increased glycine cleavage would course increase the availability of C1 units, but it would also decrease the concentration of glycine, which would in turn relieve the inhibition of SHMT and decrease the serine concentration [32]. Therefore, further enhancing GCV would not lead to improve the serine from glycine when glycine concentration was high enough.

**Fig. 5:**
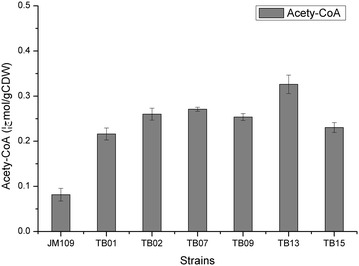
Intracellular acetyl-CoA concentrations of recombinant *E. coli*. The average cell dry weight for all of the strains was 0.38 g/l per optical-density (OD_600_) unit of culture. Histogram shows the mean of three biological replicates, and *error bars* show standard deviations

To verify that the activation of threonine bypass pathway was functional and the increased PHB production was resulted from increased acetyl-CoA, the intracellular acetyl-CoA concentration in various strains were determined. As shown in Fig. 5, deregulation of threonine degradation in TB01 increased the intracellular acetyl-CoA level by approximately threefold as compared with JM109, and activating the serine degradation and overexpressing threonine biosynthetic pathway could further improve the acetyl-CoA level. Interestingly, overexpression of *ppc* gene in TB09 caused little decrease of acetyl-CoA concentration compared with TB07. Thus, together with the increased PHB production and intracellular acetyl-CoA level, it was concluded that the threonine bypass was functional in improving intracellular acetyl-CoA in *E. coli*.

### Activating transhydrogenase for improving PHB production

Previous researches have shown that maintaining high levels of NADPH and acetyl-CoA play an important role in the PHB production [13]. We have constructed the threonine bypass to improve the intracellular acetyl-CoA for PHB production. As mentioned in the method section, the calculated optimal pathway is based on the assumption of free conversion between NADH and NADPH by transhydrogenase. However, the transhydrogenase (encoded by *pntAB*) activity in *E. coli* may be not high enough to produce NADPH from NADH generated in the EMP pathway. The availability of NADPH could be a limiting factor for PHB production after improving the acetyl-CoA levels.

In *E. coli*, 35–45 % of NADPH required for biosynthesis was produced via *pntAB* during aerobic conditions, whereas glucose 6-phosphate-1-dehydrogenase (G6PDH) and isocitrate dehydrogenase (ICD) contribute 35–45 and 20–25 %, respectively [33]. G6PDH and ICD catalyzed reactions are not in the calculated optimal pathway shown in Fig. 1 as CO_2_ is produced in these reactions, causing carbon loss. To provide enough NADPH required in threonine bypass, we overexpressed *pntAB* in the chromosome via the insertion of regulatory part Trc-162 in TB13, creating strain TB17. The *pntAB* gene transcription levels of strain TB17 were compared with parent strain TB13 through RT-PCR analysis (Additional file 1: Figure S2), suggesting that the genes have been successfully overexpressed. As shown in Fig. 4, TB17 (pBHR68) produced 6.82 g/L PHB with a content of 78.9 %. Dry cell weight was significantly increased but PHB content was decreased slightly compared with that of TB13 (pBHR68). The yield of PHB in TB17 (pBHR68) was 0.36 g/g glucose, which was almost 3.5-fold higher than that of JM109 (pBHR68). These results suggested that improvement of the availability of NADPH via engineering transhydrogenase could significantly increase the PHB production after activating the threonine bypass which requires reducing power for carbon fixation.

### Evaluation of the recombinant *E. coli* strains for the PHB production in fed-batch fermentation

**Fig. 6:**
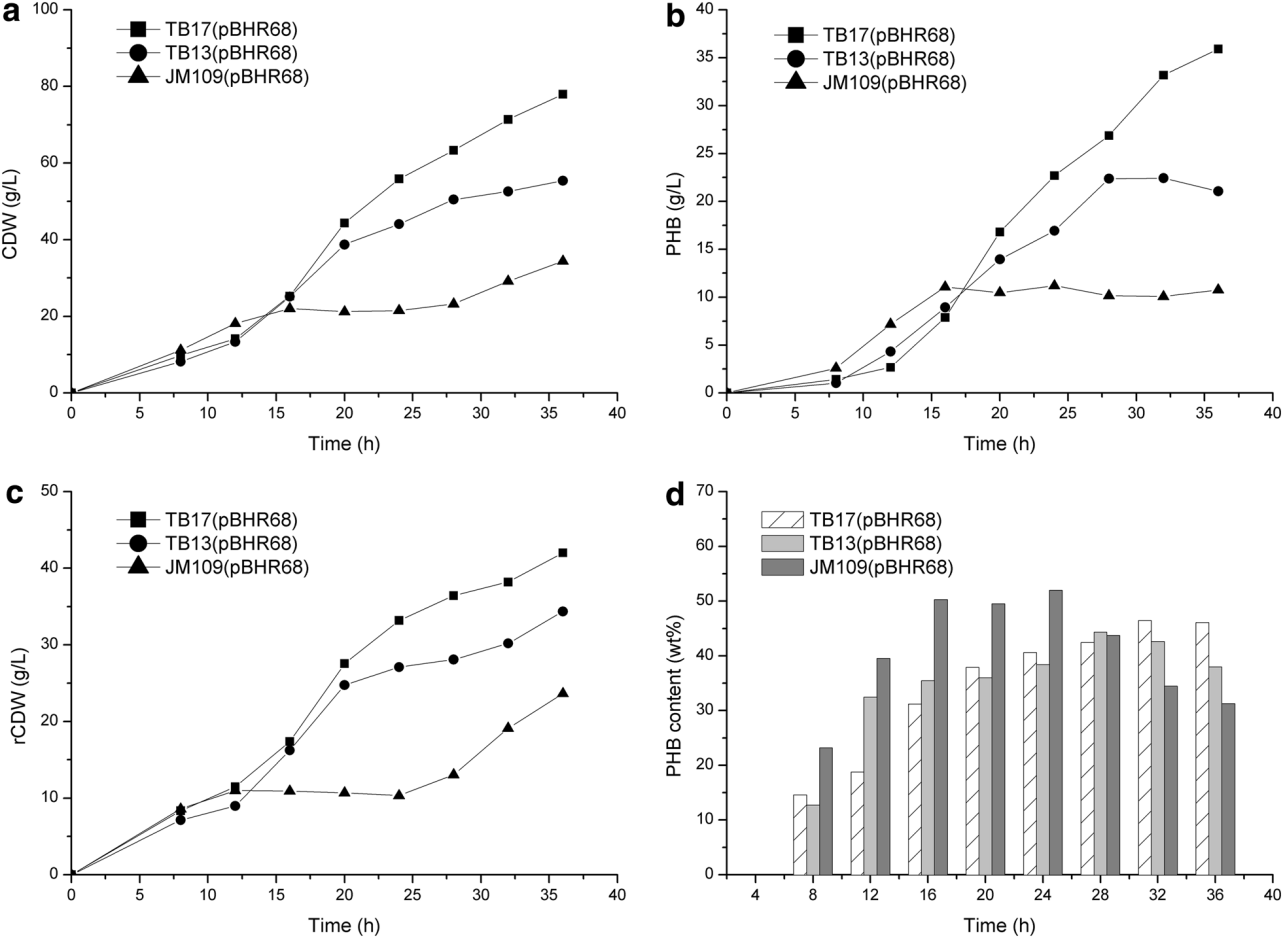
Time profiles of CDW (**a**), PHB production (**b**), residual biomass (**c**), and PHB content (**d**) of recombinant *E. coli* harboring *phbCAB* genes cultivated in a 5.0 L fermenter

To investigate the PHB production after activating the threonine bypass, fed-batch fermentation of strains TB17 (pBHR68), TB13 (pBHR68) and JM109 (pBHR68) were performed in a 5 L fermenter. The culture profile of the recombinant strains on glucose was depicted in Fig. 6. During the initial period of about 0–16 h, cell growth and the PHB production were no significant differences among these strains. During this phase, JM109 (pBHR68) had the highest PHB content among this strains (Additional file 1: Figure S3d). After 16 h, The PHB level of TB17 (pBHR68) and TB13 (pBHR68) were elevated at different time points. The final PHB titer of TB17 (pBHR68) was 35.92 g/L with a PHB content of 46.1 %, which was 3.34-fold higher compared to the parent strain JM109 (pBHR68) (10.75 g/L). TB13 (pBHR68) accumulated 22.42 g/L (PHB content 42.62 %) and thus 2.09-fold higher as compared to the reference strain JM109 (pBHR68). It appears that the threonine bypass activated strains were more productive than the control strain. The PHB yields of TB17 (pBHR68) and TB13 (pBHR68) were 0.23 g PHB/g glucose and 0.16 g PHB/g glucose, respectively, significantly higher than 0.12 g PHB/g glucose of JM109 (pBHR68). These results suggest that the engineering of threonine bypass is an effective way for increasing PHB yield as predicted by metabolic network analysis. However, the PHB yield is still lower than that obtained in the shake flask fermentation and much lower than the theoretical value. Further researches on process development and optimization are needed to achieve high PHB productivity and yield in fermenter conditions.

## Conclusions

Genome scale metabolic network analysis is an effective method to find novel metabolic engineering strategies to construct optimal strains with high productivity and yield. The present work reported the discovery of threonine bypass as a strategy for improving PHB production in *E. coli*. The engineered strain with activated threonine bypass produced 6.82 g/L PHB with the yield of 0.36 g PHB/g glucose in the shake-flask fermentation and 35.92 g/L PHB with the yield of 0.23 g/g glucose in the fed-batch fermentation, which was almost 3.3-fold higher than the parent strain. Threonine bypass, as a general pathway for CO_2_ fixation using excess reducing power and ATP, has the potential to be used as a common metabolic engineering target for a wide range of bioproducts.

## Methods

### Computational procedure

The *E. coli* metabolic network model iJO1366 was downloaded from BiGG database and loaded into the COBRA Toolbox for the calculation of optimal pathways for PHB production [34]. The glucose consumption rate was set at 10 mmol/gDCW/h for the calculation of the theoretical PHB yield of the optimal pathway NAD(P)^+^ transhydrogenase reaction is in the model and therefore NADH and NADPH were regarded as equal in the calculation.

### Strains, mediums, and cultivation conditions

All strains used in this study are summarized in Table 2. *E. coli* DH5α was used for plasmid construction. During strain construction, cultures were grown at 30 or 37 °C in Luria–Bertani (LB) broth (per liter: 10 g tryptone, 5 g yeast extract, 10 g sodium chloride) and supplemented with antibiotics as appropriate.

**Table 2:** Strains and plasmids used in this study

Strain or plasmid	Relevant genotype	Source or references
Strains		
*E. coli* DH5α	*Coli* genetic stock center strain (CGSC) No. 12384	CGSC^a^
*E. coli* JM109	*recA1*, *endA1*, *gyrA96*, *thi*, *hsdR17*, *supE44*, relA1, Δ(*lac proAB*)/*F’* [*traD36*, *proAB* ^+^, *lac* ^*q*^ *lacZ*ΔM15]	TaKaRa (Dalian, China)
TB01	JM109, P_*kbl*-*tdh*_::P_*trc*_	This study
TB02	JM109, P_*kbl*-*tdh*_::P_*trc*_, P_*sdaA*_::P_*trc*_	This study
TB03	JM109, Δ*serB*	This study
TB04	JM109, P_*kbl*-*tdh*_::P_*trc*_, Δ*serB*	This study
TB05	JM109, P_*kbl*-*tdh*_::P_*trc*_, P_*sdaA*_::P_*trc*_, Δ*serB*	This study
TB06	JM109, *thrA*(C1034T), P_*thrABC*_::P_Trc-162_	
TB07	JM109, P_*kbl*-*tdh*_::P_*trc*_, P_*sdaA*_::P_*trc*_, *thrA*(C1034T), P_*thrABC*_::P_Trc-162_	This study
TB09	JM109, P_*kbl*-*tdh*_::P_*trc*_, P_*sdaA*_::P_*trc*_, *thrA*(C1034T), P_*thrABC*_::P_Trc-162_, P_*ppc*_::P_*tac*_	This study
TB13	JM109, P_*kbl*-*tdh*_::P_*trc*_, P_*sdaA*_::P_*trc*_, *thrA*(C1034T), P_*thrABC*_::P_Trc-162_, P_*ppc*_::P_*tac*_, P_*glyA*_::P _Trc-162_	This study
TB14	JM109, P_*kbl*-*tdh*_::P_*trc*_, P_*sdaA*_::P_*trc*_, *thrA*(C1034T), P_*thrABC*_::P_Trc-162_, P_*ppc*_::P_*tac*_, P_*gcv*_::P _Trc-162_	This study
TB15	JM109, P_*kbl*-*tdh*_::P_*trc*_, P_*sdaA*_::P_*trc*_, *thrA*(C1034T), P_*thrABC*_::P_Trc-162_, P_*ppc*_::P_*tac*_, P_*glyA*_::P_*trc*_, P_*gcvA*_::P _Trc-162_	This study
TB17	JM109, P_*kbl*-*tdh*_::P_*trc*_, P_*sdaA*_::P_*trc*_, *thrA*(C1034T), P_*thrABC*_::P_Trc-162_, P_*ppc*_::P_*tac*_, P_*glyA*_::P _Trc-162_, P_*pntAB*_::P _Trc-162_	This study
Plasmids		This study
pBHR68	pBluscript SK(−) derivative, *phbA*_*Re*_, *phbB*_*Re*_, *phbC*_*Re*_ cloned from *R. eutropha*	[8]
pTKRED	pSC101 replication, temperature sensitive replication origin, *Spc* ^*r*^, P_*araBAD*_-driven I-SceI gene, Red recombinase expression plasmid, lac-inducible expression	[40]
pTKS/CS	p15A replication, *Cm* ^*r*^, *Tet* ^*r*^, I-SceI restriction sites	[40]

*Amp* ampicillin, *Cm* chloramphenicol, *Tet* tetracycline, *Spc* spectinomycin, *r* resistance

^a^Coli genetic stock center

For PHB production, minimal sodium medium (MS medium) with 1 g/L yeast extract and 20 g/L glucose was used as seed culture and shake flask medium which contained (in grams per liter): (NH_4_)_2_SO_4_ 2.0, MgSO_4_·7H_2_O 0.4, Na_2_HPO_4_ 3.83, KH_2_PO_4_ 1.5, Fe(III)-NH_4_-citrate 0.05, CaCl_2_ 0.02, and 1 mL/L trace element [35]. When necessary, a final concentration of 100 μg/mL ampicillin was added to the medium to maintain the stability of the plasmids. Colonies were inoculated into 5-mL LB culture medium and grown at 37 °C with shaking overnight. Then inoculated with 1 % into 250-mL flask as seed culture grown at 37 °C in MS medium for 12 h at 220 rpm on a rotary shaker. Seed culture was then inoculated into 500-mL flask with 100 ml culture medium (with the initial OD_600_ of 0.04) and grown at 37 °C 220 rpm on a rotary shaker for 48 h. For amino acid accumulation, MS medium with 1 g/L yeast extract and 10 g/L glucose was used.

Fed-batch cultures were incubated at 37 °C in a 5 L fermenter (Shanghai Bailun Bio. Co. Ltd.) containing 2.5 L MS medium (10 g/L yeast extract) with an initial OD_600_ of 0.05. When the glucose concentration in the culture broth fell to 1 g/L, 100 mL of 500 g/L glucose was added. Fermentations were performed at 37 °C with the pH automatically controlled at 7.0 using a 25 % solution of ammonium hydroxide. Dissolved oxygen was maintained at ≥30 % by adjusting agitation speed.

### Genome engineering

The DNA fragment insertion or replacement strains were constructed by using the method reported by Lin and co-workers [36]. The fragment construction needed three rounds of PCR. In the first round, primer U-F/U-R and L-F/L-R were used to amplify the up and low homologous arms of target site from the *E. coli* genome; the overlapping marker fragments “tetA-U” and “tetA-L” of the tetracycline resistance gene *tetA* were amplified respectively from pTKS/CS using T2/T-F and T1/T-R primers. In the second and third round PCR, the fragment was conducted by SOE-PCR. The final fragments (100 ng of purified PCR fragment) were transformed into the competent cells with expression of the λ-red recombination enzymes. The tetracycline resistant mutants were screened and confirmed by colony PCR. To induce I-SceI endonuclease expression and remove the resistance gene *tetA* from the genome, the positive colony was inoculated into 5 ml of LB medium with 100 μg/mL spectinomycin, 2 mM Isopropyl-β-D-thiogalactopyranoside (IPTG), and 0.2 % w/v l-arabinose. After overnight cultivation, cultures were diluted to appropriate concentration and plated on LB agar plates. The loss of *tetA* was confirmed by colony PCR. The technological process in detail was displayed in Additional file 1: Figure S1. Primers used were listed in Additional file 1: Table S1.

For swapping the promoters of *glyA, kbl*-*tdh* operon, *thrABC* operon, the *tetA* fragment was amplified from strain Tet-Trc-162, and fused with the up and low homologous arms. Fragment Trc-162 consisted of the *trc* promoter core sequence and M1-162 [37] in tandem.

### qRT-PCR

The recombinant strains were cultured in MS medium with 1 % (W/V) glucose. RNAs were extracted from exponentially growing cells in baffled flasks using the RNAprep pure Cell/Bacteria Kit (Tiangen, Beijing, China) as described by the manufacturer. The cDNA was amplified using FastQuant RT Kit (Tiangen, Beijing, China) with the total mRNA as the templates. Samples were then analyzed using a Light Cycler^®^480 II (Roche, Basel, Switzerland) with RealMasterMix (SYBR Green I) (Tiangen, Beijing, China). Quantity real-time PCR amplification primers were listed in Additional file 1: Table S1; they exhibited identical calculated annealing temperatures and resulted in product sizes of approximately 50–200 bps. For data analysis, the *rrsA* gene was selected as internal standard for normalization between samples and three biological replicates were performed. The obtained data were analyzed by using the 2^−ΔΔCt^ method according to described previously [38].

### Analytical techniques

The growth of cell was monitored by measuring the OD_600_ with an ultraviolet spectrophotometer (Beijing Puxi Universal Co Ltd). Glucose in the fermentation broth was determined utilizing a SBA sensor machine (Institute of Microbiology, Shangdong, China). Bacteria were harvested by centrifugation at 8000×*g* for 10 min and then washed with distilled water. Cell dry weight (CDW) was measured after vacuum lyophilization. PHB content was analyzed by gas chromatography (Persee, China) with an Agilent J&W Capillary GC column after methanolysis of lyophilized cells in chloroform.

For the determination of intracellular acetyl-CoA, 40 mL mid-exponential phase cell culture was taken into precooled centrifuge tubes and centrifuged at 8000×*g* and 4 °C for 10 min. The cell pellets were washed with 40 mL 100 mM Tris–HCl buffer (pH 8.0). Acetyl-CoA was analyzed by HPLC as previous reported [39].

## Additional file

10.1186/s12934-015-0369-3 Primer sequence used for genome manipulation in this study. **Figure S1**: Strategies for chromosomal replacement. **Figure S2**: Results of relative transcriptional level. **Figure S3**: Versions of *serB* ^-^ strain overexpressing combinations of native *kbl*-*tdh* and *sdaA* genes were tested for their ability to grow on glucose minimal medium.
