# Supplementary material for: Metabolic engineering of Escherichia coli for poly(3-hydroxybutyrate) production via threonine bypass
Source: Microb Cell Fact. 2015 Nov 20;14:185. doi: 10.1186/s12934-015-0369-3 (PMC4654888; doi:10.1186/s12934-015-0369-3)
Supplement: Supplementary file 1 — 10.1186/s12934-015-0369-3 Primer sequence used for genome manipulation in this study. Figure S1: Strategies for chromosomal replacement. Figure S2: Results of relative transcriptional level. Figure S3: Versions of serB - strain overexpressing combinations of native kbl-tdh and sdaA genes were tested for their ability to grow on glucose minimal medium. [file 12934_2015_369_MOESM1_ESM.docx]

**Supplementary Methods**

**Supplementary table S1.** Primer sequence used for genome manipulation in this study

| Primer name | Primer sequence (5’→3’) |
| --- | --- |
| **Primer used for genome manipulation** | |
| *kbl*p U_F | AAATAATAACATTGTGGACGA |
| *kbl*p U_R | AATGAGCCGGATGATTAATTGTCAACAGCTCATCAACACCTACAGATAGTAGA |
| *kbl*p L_F | ATAATGTGTGGTCACACAGTCGACGGAGTAACGACTCTCGTAAATAAGGAGTTTAAAATGCGTGGAGAATTTTATC |
| *kbl*p L_R | CGCAAATAAAACGCACCGAA |
| *SdaA*p U_F | TATACCGCCTTCCGCCGTTG |
| *SdaA*p U_R | AATGAGCCGGATGATTAATTGTCAACAGCTCATTAGTCAGGGTTTCACACCAA |
| *SdaA*p T_F | ATGAGCTGTTGACAATTAATCATCCGGCTCATTACCCTGTTATCCCTACTAAGC |
| *SdaA*p T_R | TACTCCGTCGACTGTGTGACCACACATTATACGAGCCGGATGATTAATTGTCAACAGCTCATTAGGGATAACAGGGTAATGTACC |
| *SdaA*p L_F | ATAATGTGTGGTCACACAGTCGACGGAGTAACGACTCTCGTAAATAAGGAGTTTAAAGTGATTAGTCTATTCGACAT |
| *SdaA*p L_R | AAACCGGGAATACTGTCGAT |
| *glyA*p U_F | AGCCCTGCAATGTAAATGGTT |
| *glyA*p U_R | GAGCCGGATGATTAATTGTCAACAGCTCATCCGCATCTCCTGACTCAGCT |
| *glyA*p L_F |  |
| *glyA*p L_R | TGCGCCAGGTTCATACCCAGA |
| Tet_F | AGCTGAGTCAGGAGATGCGG |
| Tet_R | AGCTGTTTCCTGGTTTAAAC |
| *gcv* U_F | ATAAACTAATTTCACCTCCGTT |
| *gcv* U_R | CCGCATCTCCTGACTCAGCTCTTGTCCTCATTGAATAAGCG |
| *gcv* L_F | GTTTAAACCAGGAAACAGCTATGGCACAACAGACTCCTTT |
| *gcv* L_R | GGCATTCAACATCCCCGAGT |
| *thrA*m U_F | GAGCTTTCCTACTTCGGCGCTA |
| *thrA*m U_R | ACATTACCCTGTTATCCCTACAGCACCACGAAAATACGGG |
| *thrA*m T_F | TATTTTCGTGGTGCTGTAGGGATAACAGGGTAATGTACCA |
| *thrA*m T_R | ATACGGGCGCGTGACATATTACCCTGTTATCCCTACTAAGCA |
| *thrA*m L_F | TAGTAGGGATAACAGGGTAATATGTCACGCGCCCGTATTTTCGTG |
| *thrA*m L_R | TTCGCCGAGATCCCACGCAAG |
| *thrABC*p U_F | TGACATAAAACTGGTCGACT |
| *thrABC*p U_R | AACAGCTCATCCGCATCTCCTGACTCAGCTGGATGTTGTGTACTCTGTA |
| *thrABC*p L_F | TACTTCAATTTGTTTAAACCAGGAAACAGCTATGCGAGTGTTGAAGTTCGG |
| *thrABC*p L_R | TCTAATACGCCGGCCATAATG |
| *ppc* U_F | ACTCAAACGATGCCCAACC |
| *ppc* U_R | AACAGGGTAATTTAATTGTCAACAGCTCACGAATAAATAGCAGGAATT |
| *ppc* T_F | GCTATTTATTCGTGAGCTGTTGACAATTAAATTACCCTGTTATCCCTACTAAGC |
| *ppc* T_R | CGATGATTAATTGTCAACAGCTCACGAATAAATAGCTAGGGATAACAGGGTAATGTACC |
| *ppc* L_F | GAGCTGTTGACAATTAATCATCGGCTCGTATAATGTGTGGACCCGAGCATATTCGCGCC |
| *ppc* L_R | ATCACTTCCGGGTTGCTG |
| *pntAB* U_F | TCAGTCTTTATCCGGCGTTC |
| *pntAB* U_R | GTCAACAGCTCATCCGCATCTCCTGACTCAGCTTTCCATCGGTTTTATTGATG |
| *pntAB* L_F | GTACTTCAATTTGTTTAAACCAGGAAACAGCTGGGAATATCATGCGAATTGG |
| *pntAB* L_R | AAACTCACCAGCGTTGTCC |
| **Primers for RT-PCR** | |
| RTthrA_F/R | CGAAGTGGATGGTAATGA/ GGCTGATAATAGTGGCTA |
| RTthrB_F/R | GCATTGCTCGGAGATGTA/ GAGGTTGTTGAGACTGAATG |
| RTthrC_F/R | GTCGCTTTATGGCACAAATG/ TCAGAATGGTCACTGGCTTA |
| RTkbl_F/R | TTATTGATGGTGTGCGTCTGT/ GCATATCGTTGTTGGCATAGC |
| RTtdh_F/R | TGCTTGACACCATGAATC/ ATATCAGACGGCGGAATA |
| RTsdaA_F/R | CTATGAAGGCAGGTAAACAG/ CGAGTAACGCTATCCAGTA |
| RTppc_F/R | TGCGTAGTAATGTCAGTATG/ GCGTTCAAGAATGTGTTC |
| RTglyA_F/R | CGATTATGATGCCGAACTGT/ CGATGTGCTCTTCCTGAC |
| RTgcvT_F/R | TGGCGGTGTGATAGATGAC/ AACAACGAGGCGGAAGAA |
| RTgcvH_F/R | TGAACAGCGAACCGTATG / CCAGCAGTGATTCCAGTT |
| RTgcvP_F/R | TCAACTTCCAGCAGGTAA / TCCAGAAGAGAAGCAGAG |
| RTpntA_F/R | TGTCAAAGTGATTGGTTA / AACAGTTTCAGCAGATTA |
| RTrrsA_F/R | TACGACCAGGGCTACACACG/ATCCGGACTACGACGCACTT |



 **Figure S1.** Strategies for chromosomal replacement. A. Genome editing cassettes are constructed by three rounds of PCR and recombinants after the first round of recombineering were selected by tetracycline; B. In the second step, the *tetA* marker was eliminated by simultaneous induction of I-SceI and Red recombinase expression. C. Fragments for promoter replacement or insertion.





**Figure. S2.** Results of relative transcriptional level. A. The transcription level of the genes in threonine utilizing pathway; B. The transcription level of the *pntAB* genes. Histogram shows the mean of three biological replicates, and error bars show standard deviations.

**
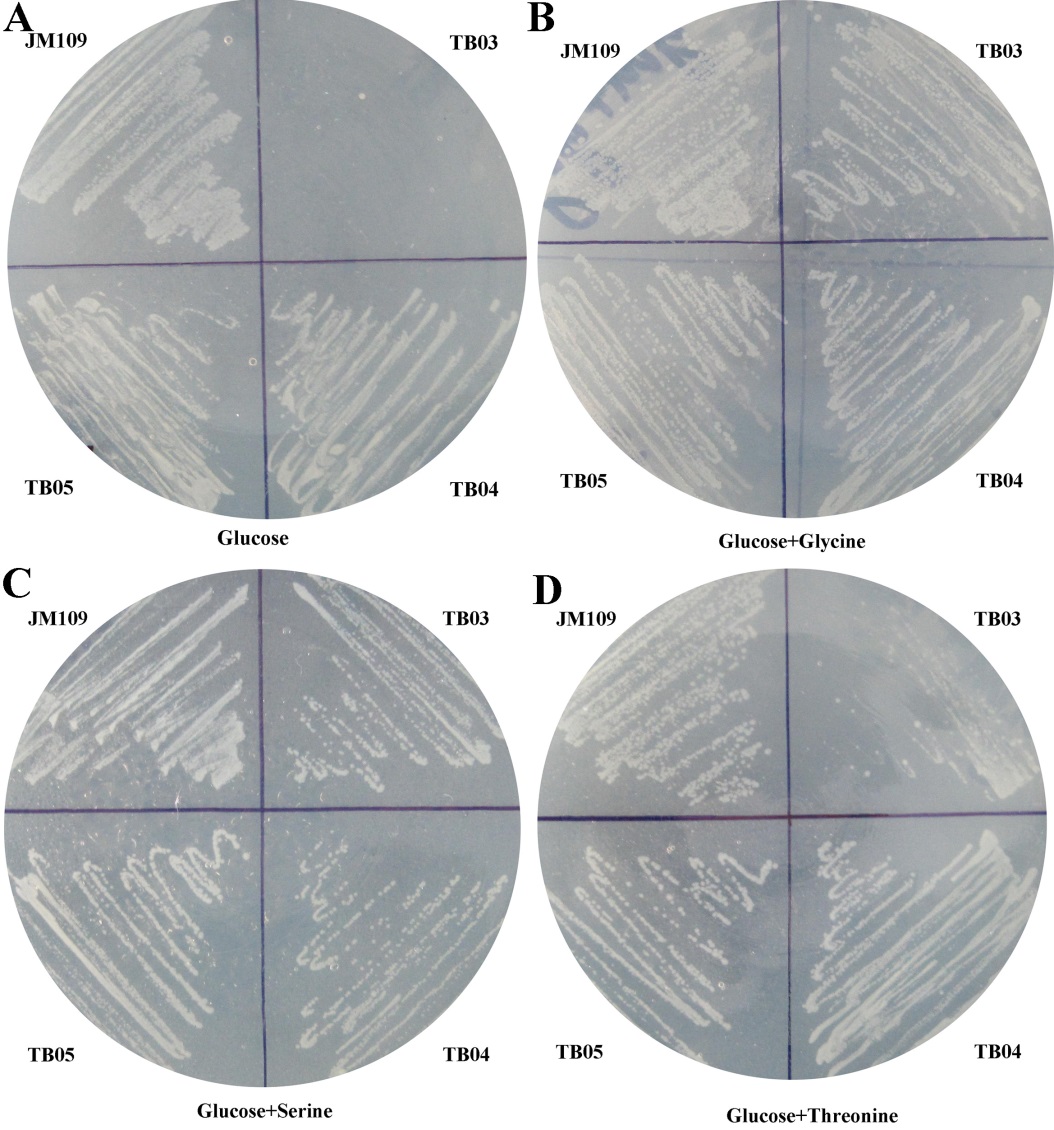
**

**Figure. S3.** Versions of *serB*^-^ strain overexpressing combinations of native *kbl-tdh* and *sdaA* genes were tested for their ability to grow on glucose minimal medium with the additives glucose (A), glucose and glycine (B), glucose and serine (C), glucose and threonine (D).
